# Anatomical Study of the Retrodural Space of Okada in the Cervical Region: 3D Micro‐CT Findings

**DOI:** 10.1002/ca.24269

**Published:** 2025-03-06

**Authors:** Tae‐Hyeon Cho, Byongnam Jun, Shin Hyung Kim, Hun‐Mu Yang

**Affiliations:** ^1^ Department of Anatomy Wonkwang University School of Medicine Iksan Republic of Korea; ^2^ Translational Research Unit for Anatomy and Analgesia Seoul Republic of Korea; ^3^ Department of Anesthesiology and Pain Medicine Yonsei University College of Medicine Seoul Republic of Korea; ^4^ Translational Laboratory for Clinical Anatomy, Department of Anatomy Yonsei University College of Medicine Seoul Republic of Korea

**Keywords:** cervical vertebrae, epidural anesthesia, micro‐CT

## Abstract

The retrodural space of Okada (RSO) is a potential space located posterior to the ligamentum flavum. It can complicate epidural injections owing to its communication with the epidural space and facet joints. The purpose of this study was to clarify the anatomical structures of the cervical RSO and adjacent ligamentous tissues in cadavers. Cervical spine specimens from 15 embalmed cadavers were used for serial sectional dissection, histological verification, and micro‐computed tomography (micro‐CT) analysis. Micro‐CT images of the RSO were acquired after phosphotungstic acid preparation at the C5‐7 levels. The RSO was examined meticulously on the basis of its three‐dimensional (3D) topography. The 3D shape of the cervical RSO was successfully visualized using micro‐CT imaging reconstruction. It had clear anatomical communications with the epidural space, facet joints, and interspinous space. Histological examination confirmed the presence of loose connective tissue within the RSO, which probably facilitates these communications. This cadaveric study demonstrated the 3D shape of the cervical RSO and its communications with adjacent anatomical structures. Further clinical studies are needed to explore the potential implications of these findings for cervical epidural injections.

## 1 Introduction

The retrodural space of Okada (RSO) is located posterior to the ligamentum flavum (LF) in the interlaminar space (Murthy et al. 2011). It is filled with adipose and loose connective tissues, allowing communication across the midline between the bilateral facet joints (Kim et al. 2023; Lawrence et al. 2021; Lehman et al. 2015). In 1981, Kikuzo Okada first described a communicating pathway from the cervical facet joint to various adjacent structures including the interlaminar portion, interspinous portion, contralateral facet joint, para‐extradural space, and cervical extradural space (Okada 1981). Researchers subsequently named this space the “retrodural space of Okada” (Jiao et al. 2019; Lehman et al. 2015; Murthy et al. 2011; Tiegs‐Heiden et al. 2022).

When the interlaminar approach is adopted for epidural injections, the loss‐of‐resistance technique is commonly used to confirm the needle's entry into the epidural space (Murthy et al. 2011; Lehman et al. 2015). However, the RSO can complicate this procedure by falsely indicating a loss of resistance, potentially misleading practitioners into believing that the needle is correctly positioned in the epidural space (Murthy et al. 2011; Reina et al. 2016). Consequently, contrast agents can spread into unintended areas such as the adjacent facet joints rather than the epidural space (Jiao et al. 2019; Kim et al. 2023; Parivash et al. 2018; Park et al. 2020).

We previously examined the structure of the RSO in the lumbar region (Kim et al. 2023). The cervical region has smaller and narrower structures, and pain‐intervention procedures in this area can lead to serious complications, including paralysis of the limbs, numbness, and thrombosis (Manchikanti et al. 2009; Nowicki et al. 2020). Therefore, it is crucial to understand the anatomical structures and histological characteristics of the RSO in the cervical region.

Technological advances have enabled the RSO to be visualized through three‐dimensional (3D) reconstructions. Traditional dissection techniques can damage the fragile tissues around the RSO, obscuring the anatomical details. Micro‐computed tomography (micro‐CT), a non‐invasive imaging method used in previous studies, provides high‐resolution 3D information and has revealed complex human tissue structures (Cho et al. 2021; Kim et al. 2023; O et al. 2019). The purpose of this study was to analyze the anatomy of the cervical RSO using 3D micro‐CT imaging, along with manual dissection and histological examination.

## 2 Methods

### 2.1 Specimen Harvest

Fifteen fixed cadavers (seven males and eight females; mean age at death, 85.9 years) were subjected to serial sectional dissection, histological verification, and micro‐CT analysis. All experimental procedures were performed ethically in accordance with the World Medical Association's Declaration of Helsinki and were approved by the Institutional Review Board of Yonsei University College of Medicine (approval no. 4‐2024‐0666). All cadavers were legally donated to the Surgical Anatomy Education Center of Yonsei University College of Medicine and were used with appropriate consent and approval (approval no. YSAEC‐24‐008). The participants provided written informed consent to donate their bodies for research after death. The authors state that every effort was made to follow all local and international ethical guidelines and laws that pertain to the use of human cadavers donated for anatomical research (Iwanaga et al. 2022).

### 2.2 Serial Sectional Dissection

Three cervical vertebral blocks were harvested from three cadavers at the C5‐7 levels. Each block was cut serially into sagittal, coronal, or axial planes at 2 cm intervals. The sectioned blocks were confirmed, and photographs and sketches were documented.

### 2.3 Histological Verification

Three tissue blocks were examined. Two of them, from two cadavers, were decalcified at the C5‐7 levels and processed for routine paraffin embedding. Longitudinal sections 5 μm thick were stained with Masson's trichrome. To obtain undecalcified horizontal sections of the cervical vertebrae at the C5 level from one cadaver, a third specimen was initially stored in 70% ethanol, then dehydrated in a series of 80%, 95%, and 100% ethanol chambers, and cleared with acetone. This specimen was infiltrated with methyl methacrylate, which was subsequently polymerized. The resulting plasticized blocks were subjected to micro‐grinding and polishing to a thickness of approximately 70 μm using an Exakt Micro Grinding System (EXAKT Technologies, Oklahoma City, OK, USA). The polished specimen was finally stained with Goldner's trichrome.

### 2.4 Phosphotungstic Acid Preparation and 3D Analysis

Nine cervical spine blocks were harvested at the C5‐7 levels from nine cadavers. All specimens were prepared in phosphotungstic acid solution according to a previously reported protocol (Cho et al. 2021; Kim et al. 2023; O et al. 2019). All specimens were scanned using SkyScan1173 (Bruker, Kontich, Belgium). The resulting data were reconstructed using NRecon (version 1.7.0.4; Bruker, Kontich, Belgium). Finally, 3D rendering was accomplished with CTvox and Materialize Mimics (version 20; Materialize NV, Leuven, Belgium), and the images were examined. All morphometric data were presented as mean ± standard deviation (SD). A parametric *t*‐test was used because the data distribution was normal. The Statistical Package of the Social Sciences (version 25.0, IBM Corporation, NY, USA) was used for all statistical analyses. A *p* value < 0.05 was considered significant.

## 3 Results

In this study, the anatomical features of the LF and the RSO were examined in all cervical vertebrae through macroscopic findings, histological observation, and micro‐CT. The LF was identified as a V‐shaped structure posterior to the dura mater that formed the dorsal wall of the vertebral canal (Figure S1). The RSO was located posterior to the LF and was connected to the facet joint between adjacent vertebrae by thin attachments, gradually thickening toward the midline.

**FIGURE 1:**
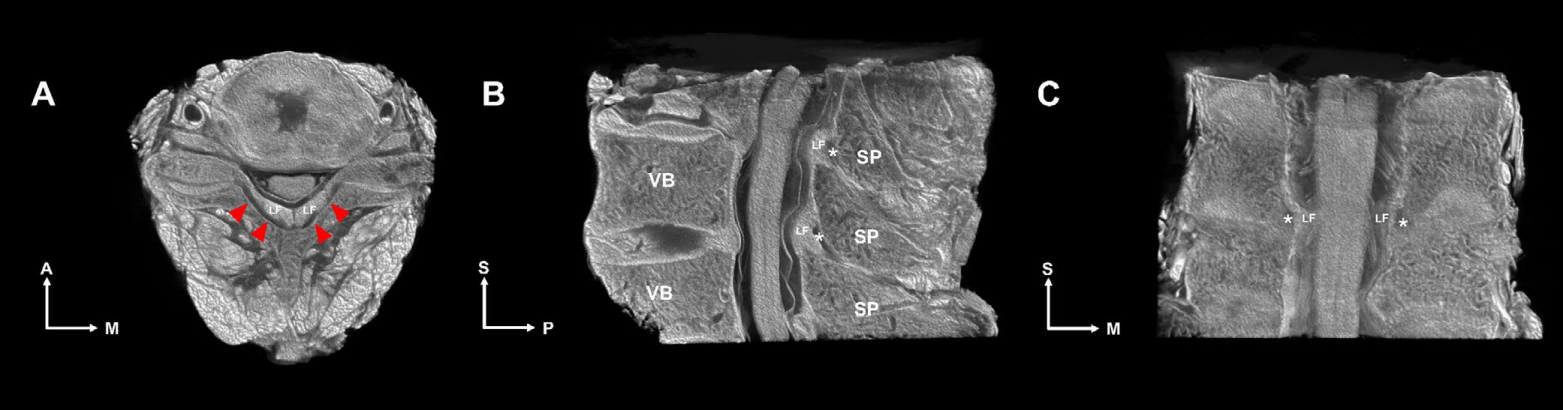
Micro‐CT images of the cervical vertebrae (C5‐7 levels) show the LF and the RSO. In the axial view (A), red arrowheads indicate the RSO, located between the LF and the vertebral lamina. The RSO connects to the epidural space anterior to the LF via a median gap. In the mid‐sagittal view (B), the RSO (indicated by an asterisk) extends posteriorly and inferiorly between adjacent spinous processes. In the coronal view (C), the RSO (indicated by an asterisk) extends to the facet joint. A, anterior; M, medial; P, posterior; S, superior; SP, spinous process; VB, vertebral body.

On axial section micro‐CT images (Figure 1 and Video S1), the RSO appeared as a distinct radiolucent space posterior to the LF and anterior to the vertebral lamina or interspinous ligament. There was a thin but distinct dehiscence (gap) between the bilateral LFs, connecting the RSO to the epidural space. The spinous process of the vertebrae extended in an infero‐posterior direction, and the RSO extended in the same direction along this process. On coronal section micro‐CT images, the RSO extended laterally to the facet joints as a tiny space. Macroscopic sections of cadaver samples corresponded with the micro‐CT findings, as shown in Figure S1.

**FIGURE 2:**
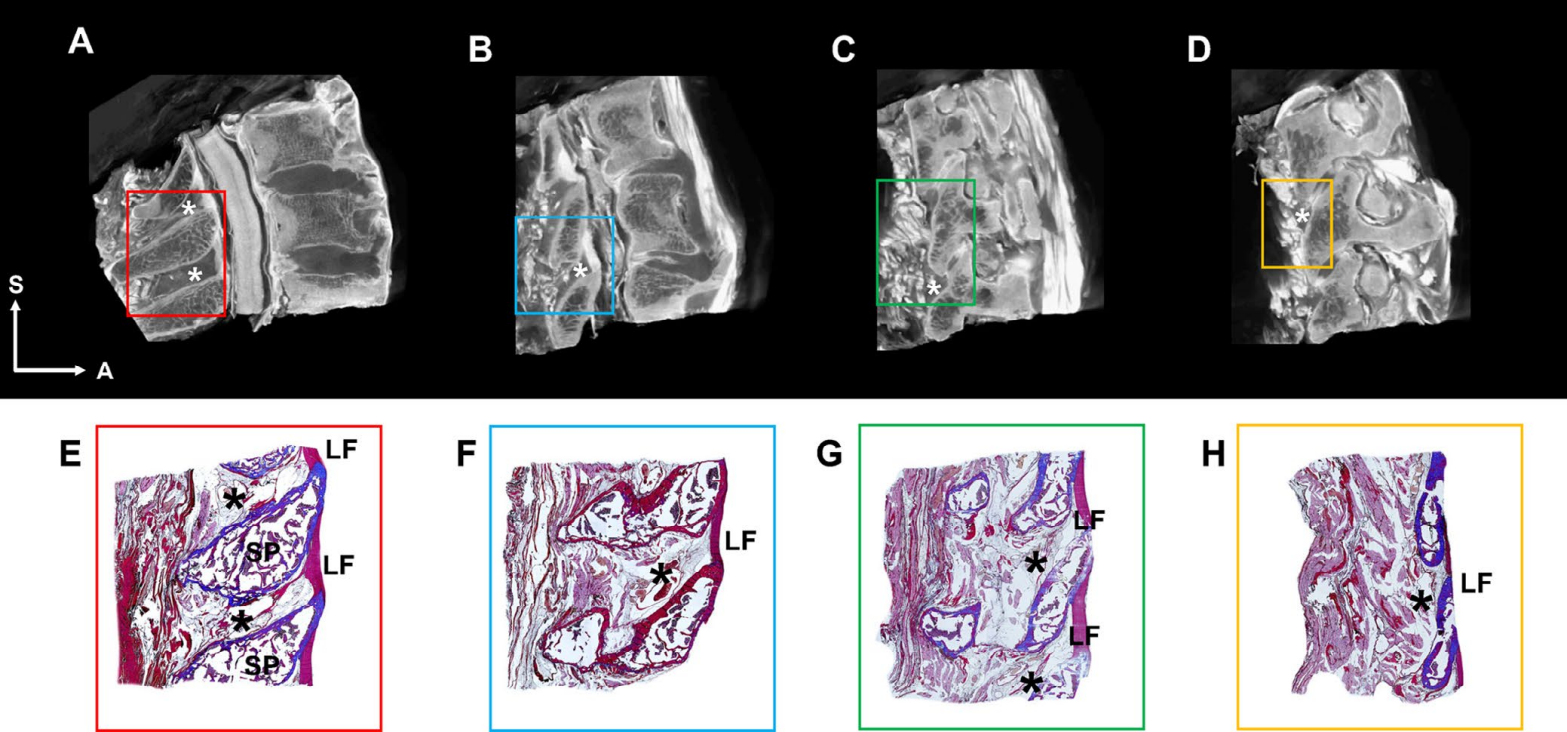
Sagittal sections of micro‐CT images (A–D) and their corresponding histological images (E–H), through the median (A, E), para‐median (B, F), lateral interlaminar (C, G), and articular (D, H) sections of the LF. There is loose connective tissue, including abundant adipose tissue, within the RSO (indicated by an asterisk). A, anterior; S, superior; SP, spinous process.

**FIGURE 3:**
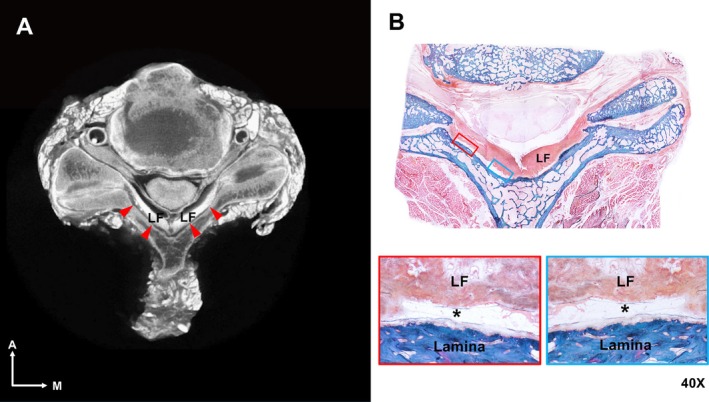
Axial sections of micro‐CT images (A) and a histological section (B). Red arrowheads indicate the RSO between the LF and the vertebral lamina. The red and blue boxes indicate the RSO (shown by an asterisk). There is loose connective tissue, including abundant adipose tissue, within the RSO.

Histological findings indicated that the RSO contained loose connective tissue, which appeared as a radiolucent space on micro‐CT images (Figure 2). The RSO extended to the facet joint and connected to the interarticular space between adjacent articular processes (Figure 3).

**FIGURE 4:**
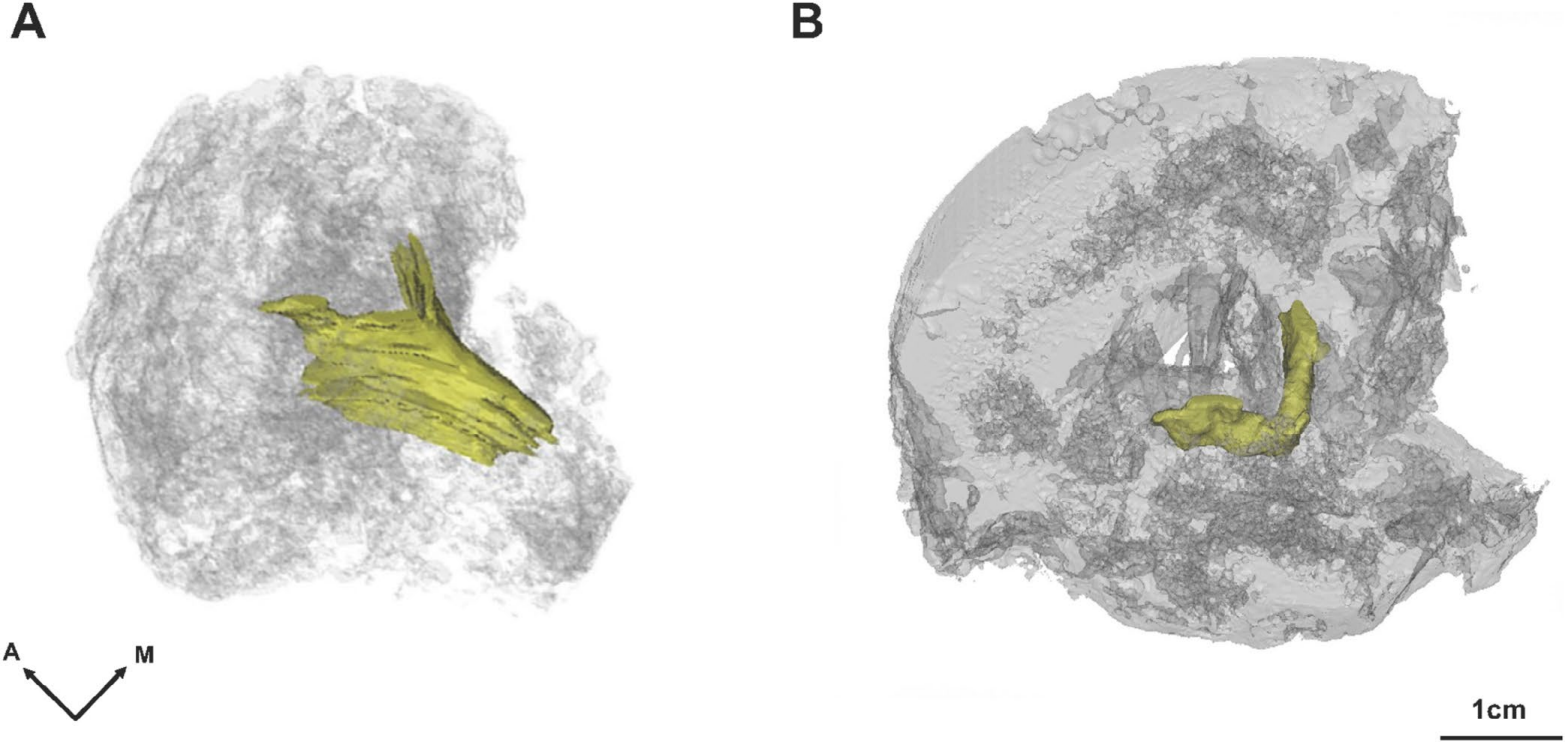
3D reconstruction image of the RSO in the cervical vertebrae (A, C5‐6 level) compared to the lumbar vertebra (B, L4‐5 level) (Kim et al. 2023). A, anterior; M, medial.

From a 3D perspective, the RSO appears as a complex space when viewed in serial sections. Therefore, a 3D virtual reconstruction of the RSO was created (Figure 4A and Figure S2) and presented in the form of a movie (Video S2) to facilitate detailed observation of continuous serial sections. As the level of the V‐shaped RSO decreased, its angle became increasingly acute.

We measured the volumes of the RSO at the C5‐6 and C6‐7 levels and compared them with each other and between genders (Table 1). The volume was 830.36 ± 118.52 mm^3^ at the C5‐6 level and 886.82 ± 120.16 mm^3^ at the C6‐7 level (*p* = 0.358). Males and females showed volumes of 912.06 ± 94.64 mm^3^ and 765.00 ± 92.23 mm^3^, respectively, at the C5‐6 level (*p* = 0.077), and 952.42 ± 90.04 mm^3^ and 831.14 ± 111.93 mm^3^, respectively, at the C6‐7 level (*p* = 0.085). The estimated volume of the RSO tended to be larger in men and at the lower cervical level, but these differences were not statistically significant.

**TABLE 1:** The volume of the retrodural space of the Okada in the cervical region.

	Male (*N* = 4)	Female (*N* = 5)	Total (*N* = 9)
C5‐6 level (range)	912.06 ± 94.64 (770.46–1033.67)	765.00 ± 92.23 (627.16–877.18)	830.36 ± 118.52
C6‐7 level (range)	952.42 ± 90.04 (833.11–1083.27)	831.14 ± 111.93 (734.82–958.59)	886.82 ± 120.16

*Note*: Data are shown as mm^3^ or mean ± standard deviation (range).

## 4 Discussion

In the present study, we visualized the 3D shape of the cervical RSO, which was first reported 40 years ago, using micro‐CT imaging reconstruction. We confirmed that the cervical RSO has anatomical communications with the epidural space and bilateral facet joints.

Our previous study identified the RSO of the lumbar vertebrae and suggested that its spatial relationship with the LF and facet joint arthropathy could contribute to the frequent injections into the RSO during the epidural approach (Kim et al. 2023). The present study showed that the cervical RSO differed in both size and shape from the lumbar RSO. The cervical RSO extends along the vertebral lamina and interspinous spaces, forming a sharper V‐shape that descends more steeply within the interspinous space than in the lumbar region. This V‐shaped morphology of the cervical RSO is probably attributable to the anatomical structure of the cervical spine, where the spinous processes slope downward more sharply than those in the lumbar region (Standring 2021). This natural curvature from the vertebral body to the spinous processes results in the characteristic V‐shape of the cervical spine, in contrast to the more horizontally oriented processes in the lumbar spine.

The cervical RSO tended to be larger in males than in females, and its volume was generally greater at C6‐7 than at C5‐6. These findings align with anatomical expectations, as male vertebral bodies tend to be larger than those of females, and vertebral dimensions generally increase at lower levels in the cervical spine (Standring 2021). However, these differences were not statistically significant. In fact, previous research has revealed no significant difference between males and females in the incidence of contrast spread into the RSO or across spinal levels during cervical epidural injections (Park et al. 2020). However, considering the small sample size in the present study, the differences in RSO size by gender and cervical level and their clinical implications for epidural injections require further anatomical and clinical studies with larger samples.

Although absolute measurements for the lumbar region are needed for confirmation, visual comparison suggests that the cervical RSO is relatively larger than the lumbar RSO (Figure 4).

Our findings concerning the continuity of the cervical RSO with the epidural space and facet joints align with the complexity of the posterior ligamentous system. Iwanaga et al. (2020) reported that the lumbar LF is not layered and is confluent with the interspinous ligament, influencing lumbar epidural procedures. Although we did not confirm a similar continuity in the cervical spine, its anatomical alignment and its larger RSO suggest a potential structural connection. This could affect the epidural needle trajectory and contrast spread during cervical procedures.

Even when vertebral size and the surrounding anatomy are considered, the cervical RSO appears much larger than the lumbar RSO. The cervical joints are used more frequently and have a wider range of motion than the lumbar joints (Cobian et al. 2011). Also, painful cervical facets are more frequently identified in patients with neck pain than in those with low back pain (Manchikanti et al. 2004). These factors could potentially contribute to making the RSO structure in the cervical spine larger than that in the lumbar spine. Furthermore, gaps in the LF midline are more common in the cervical spine than in the lumbar spine, and the space extends more posteriorly into the interspinous area, possibly contributing to the larger size of the cervical RSO (Joshi et al. 2022; Lirk et al. 2003; Yoon et al. 2014).

Even when the paramedian approach is used in cervical epidural injections, the needle's trajectory can align with the plane of the RSO owing to the anatomical orientation of this space (Furman et al. 2018). As the RSO follows the downward slope of the spinous processes in the cervical spine, its plane can run in a similar direction to the needle path during the procedure. This alignment could increase the likelihood of unintentional needle entry into the RSO, potentially leading to inadvertent spread of contrast or medication.

However, despite the larger size and anatomical alignment of the cervical RSO, unintentional RSO injections do not appear to be more frequent in clinical practice during cervical epidural procedures than in lumbar region procedures (Kim et al. 2018, 2023; Kranz et al. 2017; Parivash et al. 2018; Park et al. 2020). Previous studies have shown that contrast spreads into the facet joint during cervical epidural injections in 2.9%–6.0% of cases (Parivash et al. 2018; Park et al. 2020). In contrast, our previous study and other investigations of the lumbar region found that 3.6%–7.5% of epidural injections resulted in inadvertent contrast spread to the facet joint (Kim et al. 2018, 2023; Kranz et al. 2017). Several factors could contribute to this difference. The interlaminar space in the cervical spine, which serves as the entry to the epidural space by the needle approach, is much narrower than that in the lumbar spine, reducing the likelihood of needle tip positioning within the RSO. Furthermore, the LF of the cervical spine has smaller dimensions and is thinner than that of the lumbar spine (Rahmani et al. 2017). The lumbar spine is also more susceptible to degenerative changes caused by weight‐bearing and aging, which can increase the likelihood of communication between the epidural space and the RSO (Chokshi et al. 2010; Huang et al. 2011; Jaumard et al. 2011; Kalichman and Hunter 2007; Kim et al. 2018, 2023). However, in the cervical spine, the frequency of contrast spread to the facet joint during epidural injections was not significantly associated with prior spine surgery or degenerative conditions such as central stenosis, foraminal stenosis, or spondylolisthesis (Parivash et al. 2018; Park et al. 2020). Moreover, clinicians generally exercise greater caution during cervical procedures, which could also contribute to the lower incidence of inadvertent RSO injections.

This study has some limitations. The structural morphology of the posterior ligamentous complex is significantly influenced by the movement or position of the cervical spine. These dynamic structural variables could have been partially overlooked in this study because of the imaging modality used and the static nature of cadaver specimens. Additionally, this study used cadavers of older people, which do not necessarily provide accurate representations of younger populations or individuals with different spinal conditions. Further research encompassing a wider age range is necessary to generalize the findings.

## 5 Conclusion

This cadaveric study successfully demonstrated the 3D shape of the cervical RSO and its anatomical connections with adjacent structures such as the epidural space and facet joints. Further clinical studies to explore the implications of these findings for cervical epidural injections could assist clinicians in improving the accuracy and performance of the procedures.

## Supporting information


**Data S1.** Supplemental materials.

**Video S1.** 3D images of the RSO shown in an axial section, sagittal section, and coronal section.

**Video S2.** 3D virtual reconstruction video of the RSO.

## Data Availability

The data generated and/or analyzed during the current study are available from the corresponding author on reasonable request.
