# Supplementary material for: Anatomical Study of the Retrodural Space of Okada in the Cervical Region: 3D Micro‐CT Findings
Source: Clin Anat. 2025 Mar 6;38(6):699–705. doi: 10.1002/ca.24269 (PMC12358820; doi:10.1002/ca.24269)
Supplement: Supplementary file 1 — Data S1. Supplemental materials. [file CA-38-699-s002.docx]

**Anatomical study of the retrodural space of Okada in the cervical region: 3D Micro-CT findings**

**Supplemental Figure 1.** The axial, sagittal, and coronal sections of the cervical spine (C5–7 levels).

**Supplemental Figure 2.** A 3D reconstruction image of the RSO shown in anterior, lateral, superior, and supero-lateral views.

**Supplemental Video 1.** 3D images of the RSO shown in an axial section, sagittal section, and coronal section.

**Supplemental Video 2.** 3D virtual reconstruction video of the RSO.

**Supplemental Figure 1.** The axial (A), sagittal (B), and coronal (C) sections of the cervical spine (C5–7 levels). Asterisks indicate the RSO in all images. LF, ligamentum flavum; SP, spinous process; VB, vertebral body.

**
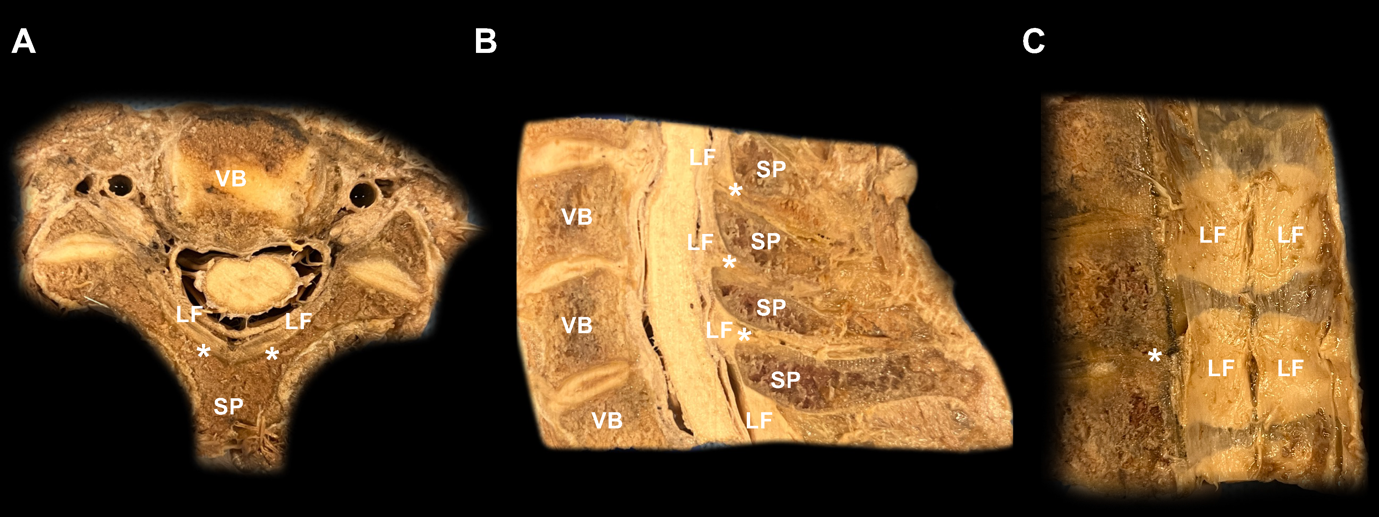
**

**Supplemental Figure 2.** A 3D reconstruction image of the RSO shown in anterior (A), lateral (B), superior (C), and supero-lateral (D) views.


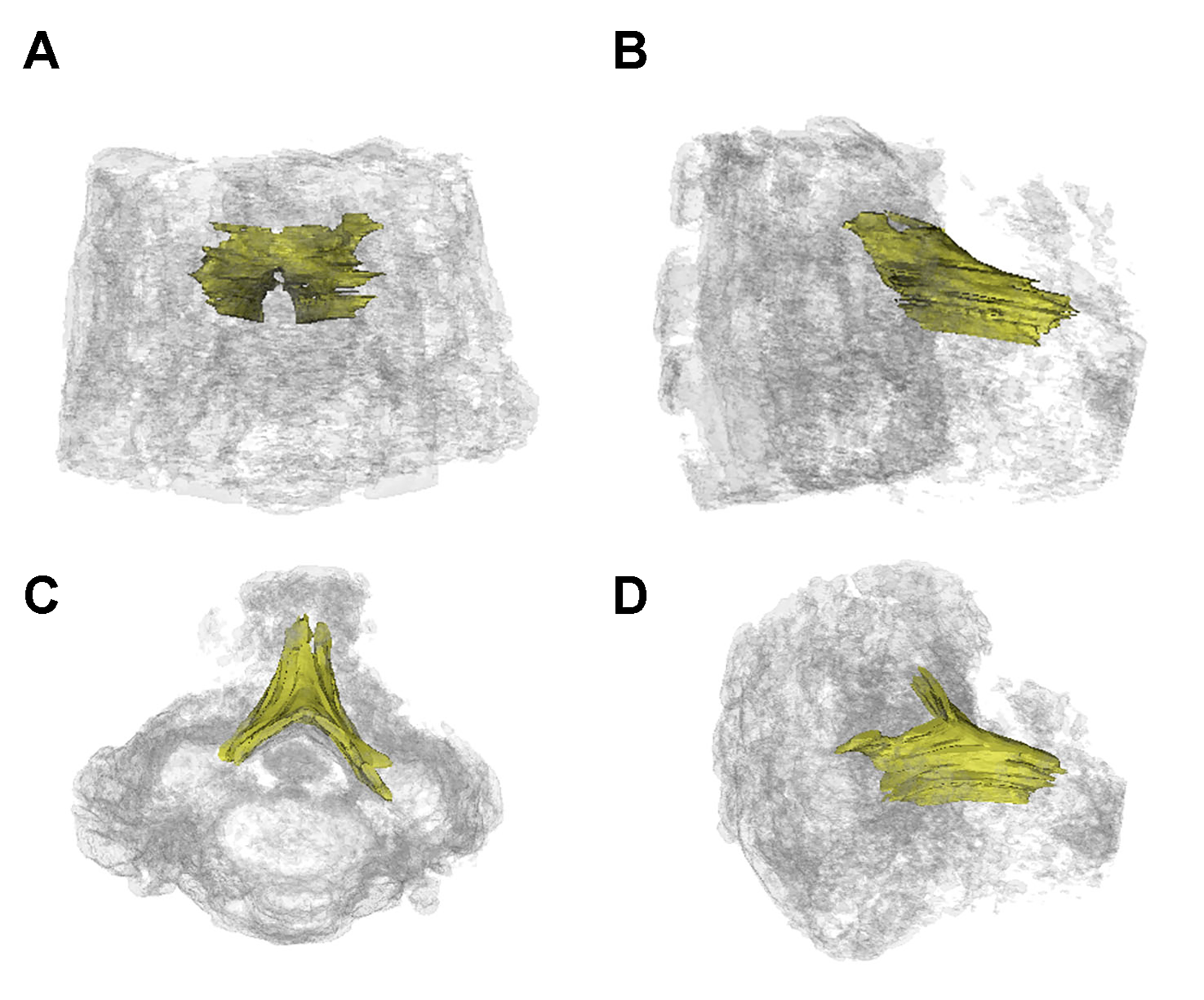


**Supplemental Video 1.** 3D images of the RSO (indicated by the red arrowheads) shown in an axial section (0:00–0:10), sagittal section (0:13–0:30), and coronal section (0:34–0:53). Sections are presented from superior to inferior in the axial section, from lateral to medial in the sagittal section, and from anterior to posterior in the coronal section, respectively. A, anterior; LF, ligamentum flavum; M, medial; P, posterior; S, superior.

**Supplemental Video 2.** 3D virtual reconstruction video of the RSO. A, anterior; L, lateral; M, medial; S, superior.
